# Evaluation of absorbent materials for use as *ad hoc* dry decontaminants during mass casualty incidents as part of the UK’s Initial Operational Response (IOR)

**DOI:** 10.1371/journal.pone.0170966

**Published:** 2017-02-02

**Authors:** Nick Kassouf, Sara Syed, Joanne Larner, Richard Amlôt, Robert P. Chilcott

**Affiliations:** 1 Research Centre for Topical Drug Delivery and Toxicology, School of Pharmacy, University of Hertfordshire, Hatfield, United Kingdom; 2 Microbial Risk Assessment and Behavioural Science, Public Health England, Emergency Response Department, Health Protection Directorate, Porton Down, Salisbury, Wiltshire, United Kingdom; National Sun Yat-sen University, TAIWAN

## Abstract

The UK’s Initial Operational Response (IOR) is a revised process for the medical management of mass casualties potentially contaminated with hazardous materials. A critical element of the IOR is the introduction of immediate, on-scene disrobing and decontamination of casualties to limit the adverse health effects of exposure. *Ad hoc* cleansing of the skin with dry absorbent materials has previously been identified as a potential means of facilitating emergency decontamination. The purpose of this study was to evaluate the *in vitro* oil and water absorbency of a range of materials commonly found in the domestic and clinical environments and to determine the effectiveness of a small, but representative selection of such materials in skin decontamination, using an established *ex vivo* model. Five contaminants were used in the study: methyl salicylate, parathion, diethyl malonate, phorate and potassium cyanide. *In vitro* measurements of water and oil absorbency did not correlate with *ex vivo* measurements of skin decontamination. When measured *ex vivo*, dry decontamination was consistently more effective than a standard wet decontamination method (“rinse-wipe-rinse”) for removing liquid contaminants. However, dry decontamination was ineffective against particulate contamination. Collectively, these data confirm that absorbent materials such as wound dressings and tissue paper provide an effective, generic capability for emergency removal of liquid contaminants from the skin surface, but that wet decontamination should be used for non-liquid contaminants.

## Introduction

Mass casualty incidents arising from the accidental or deliberate release of hazardous materials require a rapid and efficient response from emergency personnel in order to minimise the injurious effects of exposure to toxic materials. Until recently, the UK’s Model Response to chemical, biological, radiological and nuclear, or hazardous material incidents involved delaying disrobing and decontamination until the arrival of specialist response assets, such as mass decontamination units. However, recent work has demonstrated that a more rapid response is required for chemical exposures and that self-decontamination may be performed by contaminated individuals using any available absorbent material [1]. Moreover, there is evidence to suggest that showering contaminated skin with water may lead to the “wash-in effect”—the enhanced dermal absorption of particular chemicals [2–6]. In response to such practical challenges, the UK emergency services have adopted a revised process known as the Initial Operational Response (IOR). This new approach advocates dry decontamination as the default option for an emergency response [7]. A variety of bespoke decontamination products are commercially available and have been previously evaluated in our laboratory [8]. However, such products are not appropriate or practical for use in mass casualty decontamination [9].

Dry decontamination can be defined as the topical application of absorptive materials to passively remove liquid contaminants from the skin surface [10]. In theory, any absorbent material should have some degree of effectiveness for skin decontamination. However, there is a paucity of data on the clinical effectiveness of absorbent materials for the purpose of dry decontamination.

The purpose of this study was to measure the skin decontamination efficacy of absorbent materials that might be readily available on emergency vehicles (ambulances) or in clinical environments (e.g. hospital emergency departments). The current water-based protocol for clinical decontamination, known as the “rinse-wipe-rinse” (R-W-R) technique [11], was included to provide a benchmark against which the test products were evaluated.

The skin contaminants chosen for the study were selected on the basis of their historical use as chemical warfare agent simulants in human volunteer studies or dermal toxicity. Methyl salicylate and diethyl malonate are established simulants for sulphur mustard and soman, respectively [12, 13]. Parathion, phorate and potassium cyanide have demonstrable percutaneous toxicity and so were included as examples of toxic industrial chemicals [14–16].

## Materials and methods

This study consisted of two parts. First, 35 materials identified from site visits to an emergency department and ambulance station were assessed using an empirical system that measured the relative capacity of each product to absorb water and oil. Four of the test materials were then selected for evaluation regarding their efficacy in decontamination. For this purpose we used a bespoke *ex vivo* diffusion cell system containing pig skin, which has previously been described and validated for assessment of skin decontamination systems [17]. The association between the initial absorption capability of each material and its usefulness in decontamination was evaluated retrospectively.

### Chemicals and decontaminants

Radioactive (^14^C-labelled) contaminants methyl salicylate (MS), parathion (PT), diethylmalonate (DEM), phorate (PH) and potassium cyanide (KCN) were purchased from American Radiolabeled Chemicals, Inc. (ARC; Missouri, USA) and were reported to be >99% pure. Unlabelled contaminants, also with >99% purity, were purchased from Sigma Chemical Company (Dorset, UK). The radioactive liquid chemicals (MS, PT, DEM and PH) were diluted with the same unlabelled compound to achieve a working solution of 0.25 μCi μL^-1^. Similarly, ^14^C-radiolabelled and unlabelled KCN were mixed in an appropriate ratio in deionised water to produce a saturated aqueous solution (0.25 μCi μL^-1^).

Ethanol (absolute, laboratory reagent grade, >99%) and propan-2-ol (HPLC grade, 99.97%) were purchased from Fisher Scientific, Leicestershire, UK. Ultima Gold for liquid scintillation analysis and Soluene-350 for tissue digestion were purchased from Perkin Elmer, Waltham, MA, USA. Deionised water (18.2 MΩcm) was produced in house using a Milli-Q^®^ Integral 3 water purification system from Millipore.

The materials identified for initial (*in vitro*) absorbency testing were acquired either directly from an operational ambulance (Welwyn Garden City Ambulance Station, UK) or from a domestic environment (Table 1).

**Table 1:** Summary of products derived from ambulance and domestic environments for *in vitro* assessment of liquid absorbency.

Origin	Product
**Ambulance**	(1) Stretcher bedding (cotton blanket), (2) MoliNea Plus Underpad (“Green Absorbent Pad”), (3) non-proprietary white absorbent (incontinence) pad, (4) blue absorbent (incontinence) pad, (5) yellow perforated sponge, (6) triangular bandage, (7) No. 3 Dressing, (8) wound care pack dressing towel, (9) wound care pack dressing swabs, (10) Zetuvit absorbent dressing pad, (11) Bastos Viegas absorbent dressing pad, (12) Maxiflex wound dressing, (13) stretcher bedding pad, (14) Mediwrap Stretcher bedding, (15) Kimberly Clark Professional Wypall Brand L20 (“blue roll”) and (16) Ambulance Service polyurethane decontamination sponge.
**Domestic**	(17) Weetabix^™^ breakfast cereal, (18) toilet roll, (19) paper tissue, (20) kitchen roll, (21) washing-up sponge, (22) cotton bib, (23) baby wet wipes, (24) non-proprietary “super absorbent” cloth, (25) microfibre anti-grease pads, (26) “all-purpose” cloth, (27) leather wipes, (28) kitchen wipes, (29) cotton wool, (30) muslin, (31) cotton T-shirt, (32) baby nappy (diaper), (33) cat litter, (34) A4 paper, (35) cotton kitchen cloth.

For the *ex vivo* skin diffusion cell study, DEB FloraFree (detergent) was purchased from Pakex, Welwyn Garden City, UK. MoliNea Plus Underpad (“Green Absorbent Pad”), Maxiflex Dressing (manufactured by Reliance, UK), Kimberly Clark Professional Wypall Brand L20 (“blue roll”) and the Ambulance Service decontamination sponge were kindly provided by Welwyn Garden City Ambulance Station, UK.

### *In vitro* absorbency test

Circular swatches (5 cm diameter) of each test material were weighed using a fine balance (Mettler Toledo, New Classic MF model MS304S/01, Mettler Toledo Ltd., Leicester, UK) prior to being gently placed into a glass Petri dish containing ~15 mL of either MS or water. After 5 s, the swatch was removed and held above the Petri dish to allow excess liquid to flow off. The swatch was then touched against the side of the Petri dish (to remove any residual liquid) and re-weighed. The absorbance of each material was calculated as the difference between the initial weight of the swatch and the weight after contact with liquid MS or water. If the thickness of the test material was less than 0.5 cm, the material was folded repeatedly to attain a minimum thickness of 0.5 cm.

### *Ex vivo* skin absorption

Full thickness skin was obtained *post mortem* from female pigs (*Sus scrofa*, large white strain, weight range 15–25 kg) purchased from a reputable supplier. The skin was close clipped, excised from the dorsal aspect of each animal, wrapped in aluminium foil and stored flat at −20°C. Prior to the start of each experiment, skin from one animal was removed from cold storage and thawed for approximately 24 hours. Sections of the upper layer of the skin were subsequently prepared using a dermatome (Humeca Model D80, Eurogsurgical Ltd., Surrey, UK). This involved pinning out each skin section on a dissection board and drawing the dermatome over the (epidermal) skin surface using light downward pressure to produce a consistent, 1000 μm sample of tissue. This was then cut into 8 cm discs and mounted into the diffusion cells. Skin diffusion cells were manufactured by Protosheet Ltd., Kent, UK as previously described [17]. The upper (donor) chamber was modified to allow full access to the skin surface. The base of the donor chamber contained a port to allow liquid effluent to be collected, where appropriate. A fine steel mesh was used to support the skin sample, which was recessed into the base of the diffusion cell to give an area available for diffusion of 19.64 cm^2^ above a 12.5 mL flow-through receptor chamber. The receptor chamber was filled with 50% aqueous ethanol and was fed via a peristaltic pump (520S series, Watson Marlow, Cornwall, UK) at a rate of ~30 mL h^-1^. When clamped together, the top and bottom chambers could be angled through 0, 22.5, 45, 67.5 and 90° using a clamp screw and latch mechanism to allow adjustment of skin orientation where appropriate. The diffusion cells were heated using custom manufactured heat pads (Holroyd Components Ltd., Essex, UK) placed under each diffusion cell to establish a skin surface temperature of 32°C (as confirmed by infrared thermometry).

Six diffusion cells were each allocated a specific treatment. Each experiment was repeated six times, following a pseudo-Latin square design (so that no treatment was performed repeatedly in the same diffusion cell position within the fume hood), to give a total of n = 6 replicates per treatment. Treatment groups were: control (no decontamination), R-W-R decontamination method, green absorbent pad (product #2 of Table 1), Maxiflex Dressing (#12), blue roll (#15), and ambulance service decontamination sponge (#16). The latter four products were selected as being representative of the range of absorbencies measured during the prior *in vitro* studies. Each experiment was started by the addition of a 20 μL droplet of a ^14^C-radiolabelled (5 μCi) contaminant (MS, PT, DEM, PH, or KCN) to the skin surface, while it was in a horizontal position. All contaminants were added as undiluted (neat) liquid, except KCN, which was added as a saturated solution in water (0.7 g mL^-1^).

Decontamination was performed 15 minutes after skin contamination, in alignment with revised UK emergency response timescales [1]. Each of the four dry decontamination products was cut into 5 cm diameter swatches and applied directly to the skin surface. An aluminium foil disc (4.8 cm diameter) was layered onto the product and a 70 g weight (4 × 4 cm) was then placed on the foil to ensure even contact between the product and the skin surface. After 5 s, the weight and foil were removed, and the test swatch was lifted off the surface of the skin and placed into a glass vial containing 20 mL of isopropyl alcohol. The R-W-R procedure involved rinsing the skin with 10 mL detergent solution (5% DEB FloraFree), dabbing the skin surface with dry gauze, and rinsing with 10 mL tap water. The rinse procedures required the diffusion cells to be tilted until the skin was at an angle of 22.5° to the horizontal, to allow the detergent solution and water rinse effluent to be collected in pre-weighed glass vials via a port at the bottom of the donor chamber. The R-W-R procedure took ~90 s to perform. Vials containing samples of liquid effluent were reweighed and immersed in 10 mL isopropyl alcohol. The skin surface was then dried using a gauze pad, which was placed into a 20 mL pre-weighed vial with 20 mL of isopropyl alcohol.

Samples of receptor chamber fluid were collected before skin contamination (baseline) and then at 10 minute intervals after skin exposure for up to 1 hour. Each sample was collected in pre-weighed 20 mL glass vials.

At the end of the experiment, all skin surfaces were swabbed with cotton gauze to collect any residual contamination. The swabs were placed in pre-weighed vials and immersed in isopropyl alcohol (10 mL). The diffusion cells were then dismantled and the skin removed for digital autoradiography prior to immersion in Soluene-350 (50 mL) to dissolve the samples.

Autoradiography was performed using a Typhoon FLA 7000 (GE Healthcare, USA) non-confocal variable mode laser scanner. Each image comprised six skin samples (derived from the same study) and was calibrated for spatial and intensity measurements using a micro-scale slide (ARC, Missouri, USA) containing discrete areas of radioactivity ranging from 30–862 nCi g^-1^. Corresponding measurements of the intensity and area of ^14^C-contamination were assessed using ImageJ software (v1.48, U.S. National Institutes of Health, Bethesda, Maryland, USA) to provide a quantitative assessment of skin contamination (skin surface spreading).

All samples (skin surface swabs, dissolved skin and receptor chamber fluid) were analysed by liquid scintillation counting using a Perkin Elmer Tri-Carb liquid scintillation counter (Model 2810 TR), with an analysis time of 2 minutes per sample and using the manufacturer’s ^14^C-quench curve specific to the brand of liquid scintillation cocktail (Ultima GoldTM) used in this study. The amount of radioactivity in each sample was converted to a quantity of chemical by comparison to standard preparations (containing known quantities of the ^14^C-radiolabelled analogues) that were measured simultaneously.

### Data analysis

The amount of each contaminant not removed by each test product from *ex vivo* skin (%R; residual percentage of applied dose) was calculated from Eq 1.

$$\%R=\left(\frac{Q_{1}+Q_{2}+Q_{3}}{Q_{0}}\right)\times 100$$ (1)

Where Q is the quantity (μg) of radiolabelled contaminated recovered from skin surface swabs (Q_1_), solubilised skin (Q_2_) and receptor chamber fluid (Q_3_) as a proportion of the applied dose (Q_0_).

Where appropriate, a non-parametric analysis of variance (Kruskal–Wallis) was performed on the data with a Bonferroni-corrected multiple comparisons post-test (Dunn’s). Linear (Pearson) correlations were derived using GraphPad Prism (Prism version 6.04 for Windows, GraphPad Software, La Jolla California USA).

## Results

### *In vitro* absorbency

**Fig 1:**
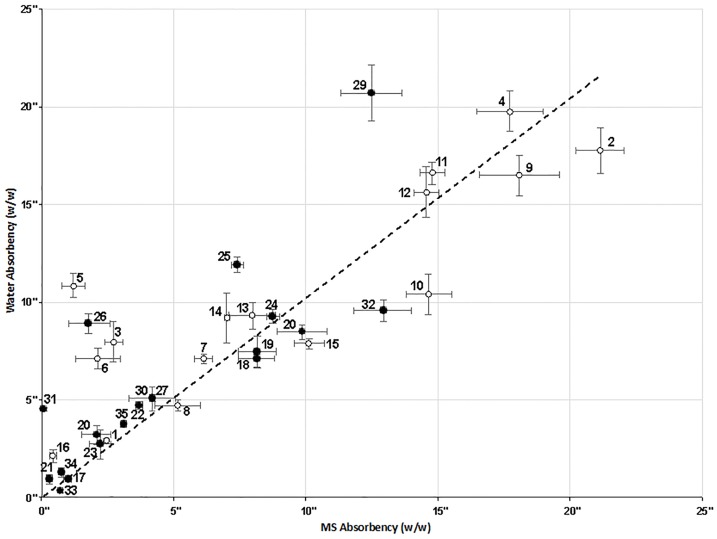
Performance of clinical (1–16; open circles) and domestic (17–35; filled circles) products in terms of ability to absorb water or methyl salicylate (expressed as weight of MS or water absorbed per weight of test product; w/w). All values are mean ± standard deviation of n = 7 measurements. Products 2, 12, 15 and 16 were selected for *in vitro* skin studies. For a description of each product (1–35), see Table 1.

There was a strong linear correlation (p<0.001) between the measured water and methyl salicylate absorbency of the test materials (Fig 1), approximating to the equation y = x (r^2^ = 0.629). There was a wide range of absorbencies (0.06–18.07 g/g); hence, four representative products, sponge (#16), blue roll (#15), Maxiflex dressing (#12) and green absorbent pad (#2), were selected for the subsequent *ex vivo* skin studies.

### *Ex vivo* studies

**Fig 2:**
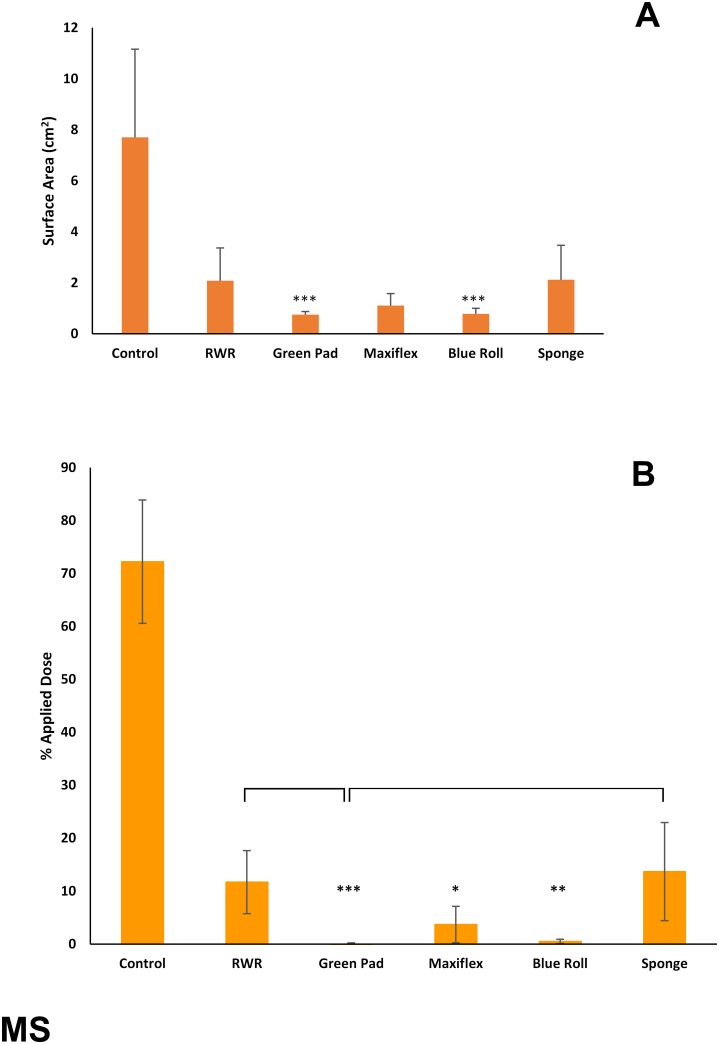
(A) Skin surface spreading and (B) recovery of ^14^C-methyl salicylate (expressed as percentage of applied dose) remaining on, within or penetrated through dermatomed pig skin following decontamination (after 15 minutes) with test products (green incontinence pads, Maxiflex wound dressing, absorbent tissue paper (blue roll) or polyurethane sponge) or the rinse-wipe-rinse method (RWR). All values are mean ± standard deviation of n = 6 replicates. Asterisks indicate significant differences between treated and untreated (control) skin: *p<0.05; **p<0.01; ***p<0.001. Horizontal brackets indicate significant differences (p<0.05) between treatment groups.

All four test products and the R-W-R method reduced skin surface spreading of ^14^C-MS by >60% (Fig 2A). This effect was statistically significant (p<0.05) for two of the test products (green pad and blue roll). Each of the test products reduced the residual amount of MS by >80% of the applied dose (Fig 2B). However, this effect was statistically significant (p<0.05) only for the green incontinence pads, Maxiflex wound dressing and blue roll tissue paper. Comparing treatments, the green incontinence pad was significantly more effective at removing ^14^C-MS than were the R-W-R method and the sponge.

**Fig 3:**
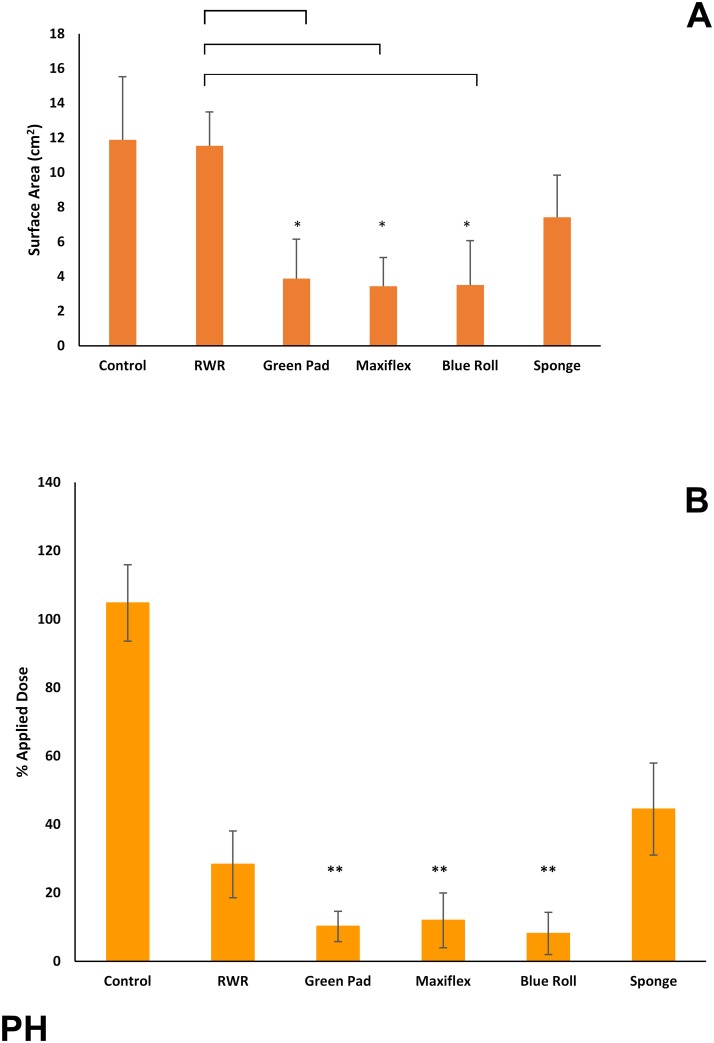
(A) Skin surface spreading and (B) recovery of ^14^C-phorate (expressed as percentage of applied dose) remaining on, within or penetrated through dermatomed pig skin following decontamination (after 15 minutes) with test products (green incontinence pads, Maxiflex wound dressing, absorbent tissue paper (blue roll) or polyurethane sponge) or the rinse-wipe-rinse method (RWR). All values are mean ± standard deviation of n = 6 replicates. Asterisks indicate significant differences between treated and untreated (control) skin: *p<0.05; **p<0.01; ***p<0.001.

The phorate results were broadly similar to those obtained with MS (Fig 3A and 3B), the two main differences being that the Maxiflex wound dressing also significantly reduced skin surface spreading of ^14^C-PH and that the R-W-R method was statistically less effective in reducing skin surface spreading than green pads, Maxiflex dressing or blue roll.

**Fig 4:**
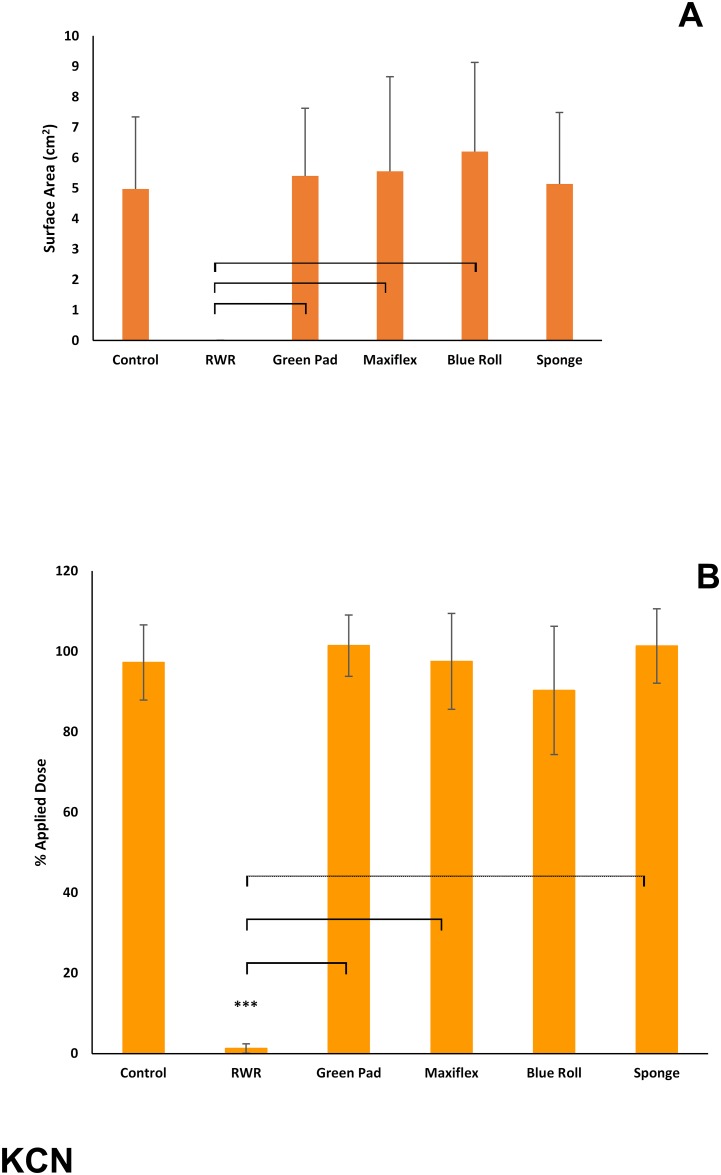
(A) Skin surface spreading and (B) recovery of ^14^C-potassium cyanide (expressed as percentage of applied dose) remaining on, within or penetrated through dermatomed pig skin following decontamination (after 15 minutes) with test products (green incontinence pads, Maxiflex wound dressing, absorbent tissue paper (blue roll) or polyurethane sponge) or the rinse-wipe-rinse method (RWR). All values are mean ± standard deviation of n = 6 replicates. Asterisks indicate significant differences between treated and untreated (control) skin: *p<0.05; **p<0.01; ***p<0.001. Horizontal brackets indicate significant differences (p<0.05) between treatment groups.

The test products were ineffective against KCN (Fig 4A and 4B). In contrast, the R-W-R method significantly (p<0.05) reduced both the skin surface spreading and the residual amounts of KCN.

**Fig 5:**
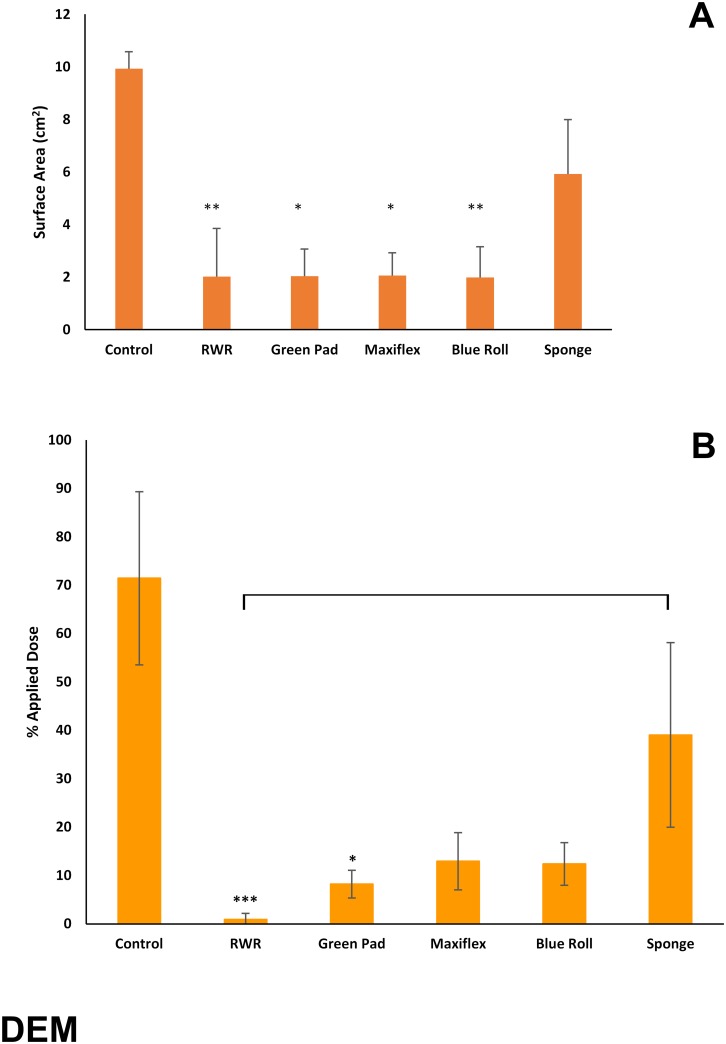
(A) Skin surface spreading and (B) recovery of ^14^C-diethyl malonate (expressed as percentage of applied dose) remaining on, within or penetrated through dermatomed pig skin following decontamination (after 15 minutes) with test products (green incontinence pads, Maxiflex wound dressing, absorbent tissue paper (blue roll) or polyurethane sponge) or the rinse-wipe-rinse method (RWR). All values are mean ± standard deviation of n = 6 replicates. Asterisks indicate significant differences between treated and untreated (control) skin: *p<0.05; **p<0.01; ***p<0.001. Horizontal brackets indicate significant differences (p<0.05) between treatment groups.

With the exception of the sponge, all test products were significantly effective in reducing the skin surface spreading and residual amounts of ^14^C-DEM (Fig 5A and 5B).

**Fig 6:**
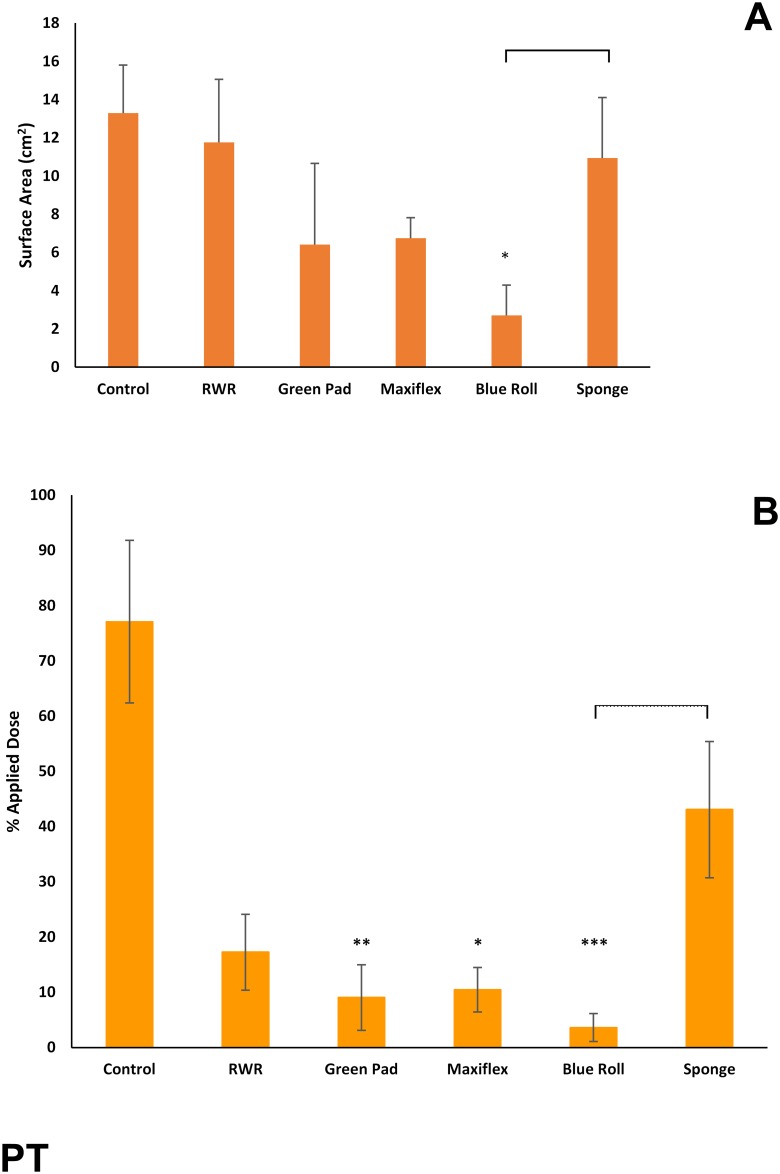
(A) Skin surface spreading and (B) recovery of ^14^C-parathion (expressed as percentage of applied dose) remaining on, within or penetrated through dermatomed pig skin following decontamination (after 15 minutes) with test products (green incontinence pads, Maxiflex wound dressing, absorbent tissue paper (blue roll) or polyurethane sponge) or the rinse-wipe-rinse method (RWR). All values are mean ± standard deviation of n = 6 replicates. Asterisks indicate significant differences between treated and untreated (control) skin: *p<0.05; **p<0.01; ***p<0.001. Horizontal brackets indicate significant difference (p<0.05) between treatment groups.

Whilst the effect was not statistically significant, green pads and Maxiflex dressing reduced the spreading of parathion by ~60% (Fig 6A). Only blue roll was statistically effective (p<0.05) in reducing the spreading of ^14^C-PT. Three test products (green pads, Maxiflex dressing and blue roll) were significantly effective in reducing residual skin contamination of PT (Fig 6B).

### Comparison of *in vitro* and *ex vivo* models

There was no significant correlation between the *ex vivo* effectiveness of the four test products (green absorbent pad, blue roll, Maxiflex wound dressing and ambulance sponge) against ^14^C-MS and their *in vitro* absorbency.

## Discussion

This study demonstrated that absorbent materials commonly found in clinical environments, such as hospitals and ambulances, are generally more effective than the standard R-W-R technique for the decontamination of liquid chemicals from the skin surface. These data are in agreement with previous *in vitro*, *in vivo* and human volunteer studies, which indicated that dry decontamination is effective for the removal of liquid chemicals [18]. Moreover, dry decontamination requires considerably less time (<5 s) than does the R-W-R method (90 s). However, the dry decontamination products were ineffective against potassium cyanide, whereas the R-W-R method was highly effective. This disparity is most probably due to the presentation of the contaminant on the skin surface: unlike the other (liquid) contaminants used in this study, potassium cyanide is a solid and so was dissolved in water for topical application. Fifteen minutes after skin exposure (at the time of decontamination), the water was observed to have evaporated, leaving a crystalline deposit on the skin surface. Thus, the experiments with cyanide reflect contamination of the skin with particulate material rather than liquid. Therefore, these data confirm that, whilst dry decontamination should be the default option for skin contaminated with liquid chemicals, aqueous decontamination is required to remove particulate contamination.

A second outcome of this study was that an *in vitro* measure of absorbency was not predictive of a test product’s *ex vivo* effectiveness as a skin decontaminant. The findings of the *ex vivo* study may be considered as robust, as they were derived from a validated skin diffusion cell model [17,19,20] that has been used extensively for assessing decontamination products [21–34]. The lack of predictive accuracy of the *in vitro* test is disappointing, as a simple method for identifying potentially effective skin decontaminants would have practical utility for the rapid screening of a wide range of products. It is conceivable that the disparity between the *in vitro* and *ex vivo* models was due to a threshold effect: any material with a degree of oil or water absorbency would be likely to demonstrate at least some ability to remove contaminants from the skin surface. Further work is required to investigate this hypothesis.

It should be noted that this study used a statistical analysis that included a mathematical (Bonferroni) correction to account for multiple comparisons. This method of analysis will substantially increase the threshold at which results are deemed significant. Whilst this is good practice for comparing multiple data sets, it represents a very conservative method of analysis. It should be considered that, whilst some of the test products showed no statistically significant effect in reducing residual skin contamination, the green absorbent pads, Maxiflex dressing and blue roll all consistently attained >90% removal of liquid skin contaminants. In contrast, the R-W-R method was ~85% effective. Thus, it is important to make a distinction between statistical significance and clinical relevance: removing 85–90% of a toxic skin contaminant will almost certainly be of some benefit to the casualty.

A final consideration is the decontamination procedure used in the *ex vivo* study. Each absorbent product was placed on the skin surface for a brief period (5 s) with slight pressure (~4.4 g cm^-2^). No attempt was made to rub the skin or perform a blotting motion. Thus, the *ex vivo* study may have underestimated the performance of dry decontaminants. Whilst this would not affect the lack of correlation observed with the *in vitro* absorbency test, it does indicate scope for further improvement in the effectiveness of decontamination.

In summary, materials that display absorbent characteristics may provide a generic capability for emergency skin decontamination. In particular, absorbent products such as wound dressings, incontinence pads and tissue paper appear to be highly effective for removing liquid contaminants from the skin surface.
